# Enhanced immunogenicity of pneumococcal surface adhesin A (PsaA) in mice via fusion to recombinant human B lymphocyte stimulator (BLyS)

**DOI:** 10.1186/1745-6150-6-9

**Published:** 2011-02-09

**Authors:** Dennis O Gor, Xuedong Ding, Qing Li, Dilara Sultana, Salamatu S Mambula, Richard J Bram, Neil S Greenspan

**Affiliations:** 1 Department of Pathology, Case Western Reserve University School of Medicine, Cleveland, Ohio 44106-4943, USA; 2 Department of Pathology, University Hospitals Case Medical Center, Cleveland, Ohio 44106, USA; 3 Beth Israel Deaconess Medical Center, Harvard School of Medicine, Boston, Massachusetts 02115, USA; 4 Department of Immunology, Mayo Clinic College of Medicine, Rochester, Minnesota 55905, USA; 5 Department of Pediatric and Adolescent Medicine, Mayo Clinic College of Medicine, Rochester, Minnesota 55905, USA; 6 Department of Surgery, Mayo Clinic College of Medicine, Rochester, Minnesota 55905, USA

## Abstract

**Background:**

B lymphocyte stimulator (BLyS) is a member of the tumor necrosis factor superfamily of ligands that mediates its action through three known receptors. BLyS has been shown to enhance the production of antibodies against heterologous antigens when present at elevated concentrations, supporting an immunostimulatory role for BLyS *in vivo*.

**Methods:**

We constructed a fusion protein consisting of human BLyS and Pneumococcal Surface Adhesin A (PsaA) and used this molecule to immunize mice. The immunostimulatory attributes mediated by BLyS *in vivo *were evaluated by characterizing immune responses directed against PsaA.

**Results:**

The PsaA-BLyS fusion protein was able to act as a co-stimulant for murine spleen cell proliferation induced with F(ab')_2 _fragments of anti-IgM *in vitro *in a fashion similar to recombinant BLyS, and immunization of mice with the PsaA-BLyS fusion protein resulted in dramatically elevated serum antibodies specific for PsaA. Mice immunized with PsaA admixed with recombinant BLyS exhibited only modest elevations in PsaA-specific responses following two immunizations, while mice immunized twice with PsaA alone exhibited undetectable PsaA-specific serum antibody responses. Sera obtained from PsaA-BLyS immunized mice exhibited high titers of IgG1, IgG2a, IgG2b, and IgG3, but no IgA, while mice immunized with PsaA admixed with BLyS exhibited only elevated titers of IgG1 following two immunizations. Splenocytes from PsaA-BLyS immunized mice exhibited elevated levels of secretion of IL-2, IL-4 and IL-5, and a very modest but consistent elevation of IFN-γ following *in vitro *stimulation with PsaA. In contrast, mice immunized with either PsaA admixed with BLyS or PsaA alone exhibited modestly elevated to absent PsaA-specific recall responses for the same cytokines. Mice deficient for one of the three receptors for BLyS designated Transmembrane activator, calcium modulator, and cyclophilin ligand [CAML] interactor (TACI) exhibited attenuated PsaA-specific serum antibody responses following immunization with PsaA-BLyS relative to wild-type littermates. TACI-deficient mice also exhibited decreased responsiveness to a standard pneumococcal conjugate vaccine.

**Conclusion:**

This study identifies covalent attachment of BLyS as a highly effective adjuvant strategy that may yield improved vaccines. In addition, this is the first report demonstrating an unexpected role for TACI in the elicitation of antibodies by the PsaA-BLyS fusion protein.

**Reviewers:**

This article was reviewed by Jonathan Yewdell, Rachel Gerstein, and Michael Cancro (nominated by Andy Caton).

## Background

B lymphocyte stimulator (BLyS, also designated TALL-1, THANK, BAFF, TNFSF13b, and TNFSF20) is a member of the tumor necrosis factor superfamily of ligands [1,2]. BLyS is expressed by activated T cells, activated macrophages, and dendritic cells [1,3,4] and has been implicated in autoimmune disorders characterized by the presence of pathological concentrations of self-antigen-reactive antibodies, such as systemic lupus erythematosus (SLE) [5] and rheumatoid arthritis (RA) [6]. Biological activity of BLyS is mediated via three receptors present on B and T cells designated transmembrane activator and calcium-modulator and cyclophilin ligand [CAML] interactor (TACI), B-Cell Maturation Antigen (BCMA) and BAFF Receptor (BR3 or BAFF-R) [7]. A functionally related molecule, designated APRIL (A Proliferation Inducing Ligand) [8] has also been described in mice and humans. APRIL binds to TACI and BCMA but not to BAFF-R [9].

Our laboratory has been interested in attempting to define mechanisms that influence the elicitation of antibody responses in the mammalian host. In this regard, we have been interested in strategies that increase the magnitude and diversity of antibody isotypes and cell-mediated immune responses to antigens of interest, while minimizing non-specific and frequently deleterious immune responses that normally accompany the use of powerful adjuvants such as complete Freund's adjuvant (CFA) and other bacterially derived products [10]. Therefore, BLyS was of considerable interest to us given numerous reports in the literature that demonstrated direct effects of BLyS on B cells. Transgenic mice that over-express the human ortholog of BLyS exhibit marked splenomegaly characterized by elevated numbers of B cells, as well as elevated concentrations of serum antibodies [11,12]. A similar, though transitory elevation in serum immunoglobulin has also been observed following daily administration of purified BLyS to mice.

One of these studies demonstrated an elevation only in serum IgM and IgA, but not IgG [1], while another study noted elevation in the serum concentrations of IgM, IgA, IgG and IgE [13]. Yet another study demonstrated that daily administration of BLyS to mice immunized with T-independent, or T-dependent antigens resulted in substantial elevations of antigen-specific serum antibody titers [14].

Collectively, these observations provided us the rationale to evaluate the ability of BLyS to act as a co-stimulant for a T-dependent antibody response *in vivo*. To that end we constructed a genetic fusion of BLyS to the test antigen PsaA (pneumococcal surface adhesin A) [15]. PsaA is one of a number of highly conserved proteins expressed by *Streptococcus pneumoniae *that are currently being investigated for possible inclusion in a third-generation protein-based pneumococcal vaccine that can extend protective coverage to capsular serotypes not represented in the currently licensed second-generation polysaccharide-diphtheria toxoid conjugate vaccines [16]. Immunity to PsaA has previously been shown to be protective against pneumococcal infection, and the development of strategies that can enhance the immunogenicity of this, as well as other pneumococcal protein antigens for possible use in future vaccines are therefore of interest.

We reasoned that optimal stimulation of immune responses might be achieved when the antigenic stimulus (PsaA) and the co-stimulatory signal (BLyS) were temporally and spatially linked. Our results indicate that fusion of BLyS to the test pneumococcal antigen PsaA, results in enhanced PsaA-specific cellular and antibody responses, compared to immune responses elicited by simple co-administration of BLyS together with PsaA or PsaA alone in the absence of conventional adjuvants. This study also reports an unexpected role for TACI in the enhanced immunogenicity of the PsaA-BLyS fusion in the immunized host.

## Results

### Expression, purification and characterization of recombinant proteins

**Figure 1:**
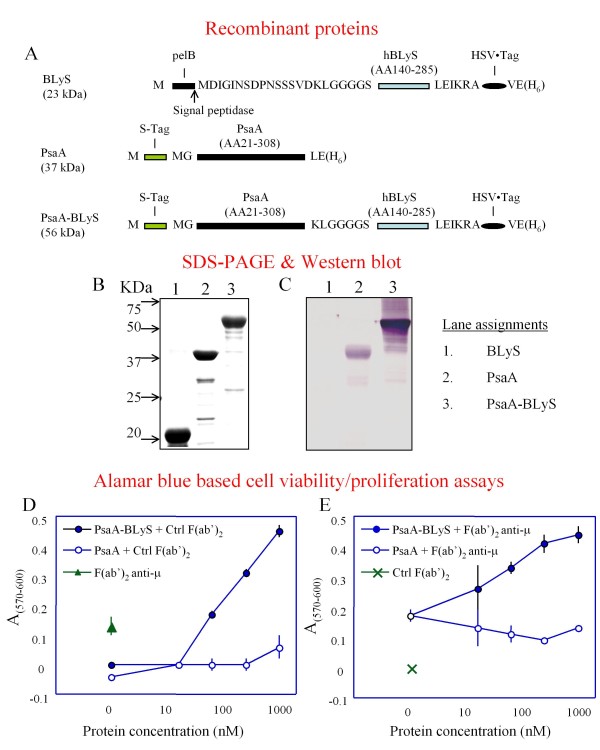
**Design, purification and characterization of recombinant proteins**. A, Diagrammatic representation of recombinant proteins purified from lysates of *E. coli*. Purified recombinant proteins were subjected to SDS-polyacrylamide gel electrophoresis and were detected by direct staining with Coomassie blue (B), or by western blot using a PsaA-specific mouse anti-serum (C). The ability of BLyS-containing fusion protein to enhance the viability of splenocytes from C3H/HeJ mice was tested in the absence (D) or presence of a suboptimal concentration of polyclonal F(ab')_2 _anti-μ (E).

We constructed a series of plasmids that were used to direct the expression of recombinant proteins in *E. coli *(Figure 1A). Recombinant proteins containing BLyS were purified from inclusion bodies following IPTG induction of the relevant *E. coli *expression strains. PsaA was purified from the soluble fraction of IPTG induced *E. coli *lysates as previously described [17]. Evaluation of the recombinant proteins by sodium dodecyl sulfate-polyacrylamide gel electrophoresis (SDS-PAGE) (Figure 1B) showed protein bands of the expected molecular masses, and western blot with PsaA-specific antisera (Figure 1C) confirmed the same. We used standard proliferation assays to test the biological activity of PsaA-BlyS. The results of 8 independent experiments performed using splenocytes from BALB/c mice and C3H/HeJ mice demonstrated that PsaA-BLyS was able to enhance the viability of splenocytes when used alone (Figure 1D), or when used in conjunction with F(ab')_2 _anti IgM fragments as a B cell proliferation stimulus (Figure 1E). These data confirmed that PsaA-BLyS retained the stimulatory [12] and co-stimulatory [1,2] activities previously described for BLyS. We were unable to demonstrate any co-stimulatory activity of BLyS when used in conjunction with suboptimal concentrations of hamster anti-mouse CD3ε to stimulate T cell proliferation. Previous experiments by others have observed BLyS-mediated T cell co-stimulation only when BLyS is immobilized on culture plates, but not in soluble form [18,19]. However, we were able to demonstrate a potent stimulation of IFN-γ secretion by splenocytes in response to BLyS together with suboptimal concentrations of anti-CD3ε, (data not shown). These data are consistent with previous observations that BLyS has T cell co-stimulatory activity [18-20].

### Antibody responses elicited by immunization

**Figure 2:**
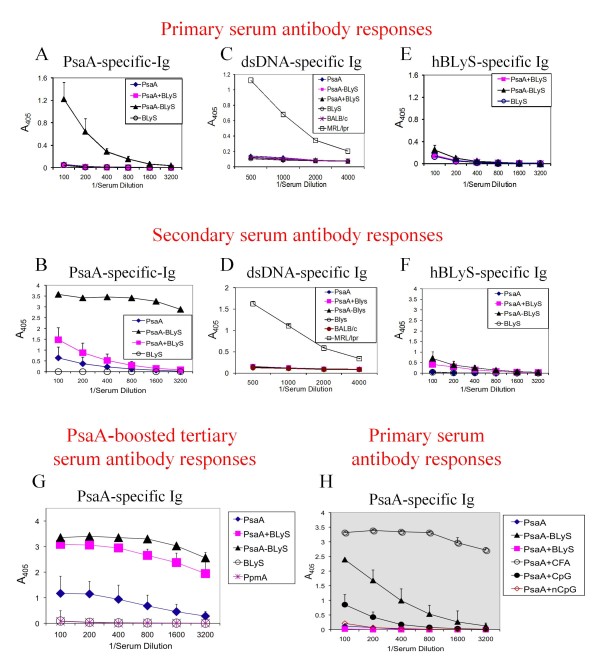
**Evaluation of antigen-specific and non-specific immunoglobulin responses**. PsaA specific serum antibody responses were determined for BALB/c mice immunized with the indicated recombinant proteins. Mice were immunized on weeks 0 and 3 and bled at weeks 2 and 5 for evaluation of primary (A, C and E) and secondary (B, D and F) antibody responses to PsaA, human BLyS and dsDNA, respectively, as indicated. Serial dilutions of individual sera from 5 mice were tested for total (Igκ) antibodies to specific antigens by ELISA and are plotted as means ± SEM for 5 mice per group. G, Mice immunized twice with the indicated antigens as in Figure 2B were boosted with PsaA alone and bled at week 9 for PsaA specific antibody responses. H, The relative immunogenicities of PsaA administered with various adjuvant formulations was tested in C3H/HeJ mice. Groups of mice were immunized once and bled after 2 weeks for the evaluation of PsaA-specific total antibody responses by ELISA. Data are presented as means ± SEM for 5 mice per group.

Previous studies in our laboratory had established that immunization of mice with PsaA at 100 pmol (3.7 μg per mouse) in the absence of adjuvants resulted in the elicitation of low to undetectable PsaA-specific antibodies. We therefore chose this concentration of protein for all immunization experiments with PsaA. Mice immunized once with PsaA exhibited the expected low to undetectable PsaA-specific serum antibody responses (Figure 2A). Mice immunized once with the PsaA-BLyS fusion protein (PsaA-BLyS) exhibited a robust PsaA-specific antibody response (Figure 2A). Mice immunized once with equimolar concentrations of PsaA and BLyS (3.7 μg PsaA + 1.9 μg BLyS, corresponding to 100 pmol of each) exhibited no elevation of PsaA specific serum antibodies (Figure 2A). Antibody responses following a second immunization of mice with the same antigen combinations (PsaA, PsaA + BLyS, or PsaA-BLyS) resulted in no PsaA-specific antibody response to PsaA immunization, a modestly elevated PsaA-specific antibody response to PsaA + BLyS, and a far more robust PsaA-specific response to PsaA-BLyS (Figure 2B).

Because mice with elevated levels of BLyS (BLyS transgenic mice, [12,21]) tend to exhibit high titers of circulating autoantibodies, we sought to evaluate the effect of immunizations with BLyS on the levels of autoantibodies in our experiments. To that end mice immunized once (Figure 2C) or twice (Figure 2D) with PsaA-BLyS, PsaA + BLyS or BLyS alone all failed to exhibit elevated dsDNA-specific antibody responses. In contrast, sera from non-immunized 6-month-old mice of the autoimmune prone MRL/lpr strain (positive dsDNA antibody control) exhibited high titers of dsDNA specific antibodies. We also observed that mice immunized once with PsaA-BLyS, PsaA + BLyS or BLyS alone exhibited undetectable human BLyS-specific antibodies (Figure 2E), while immunization twice with PsaA-BLyS or PsaA + BLyS exhibited modest but detectable human BLyS-specific antibody responses. Mice immunized twice with PsaA alone or BLyS alone had undetectable human BLyS-specific antibody responses (Figure 2F).

We subsequently used lipopolysaccharide (LPS) resistant C3H/HeJ mice to simultaneously rule out an LPS contamination effect on immune responses elicited by the recombinant proteins, and to evaluate the ability of a series of adjuvant formulations to prime for a primary PsaA-specific antibody response. As shown in Figure 2G, PsaA emulsified in Freund's complete adjuvant exhibited the most robust PsaA-specific antibody responses, followed in order of effectiveness by the PsaA-BLyS fusion protein, and PsaA admixed with CpG containing oligodeoxynucleotides (CpG). The remaining admixed combinations of PsaA + non-CpG containing oligodeoxynucleotides (non-CpG) or PsaA + BLyS were not effective at eliciting a primary PsaA-specific antibody response.

**Figure 3:**
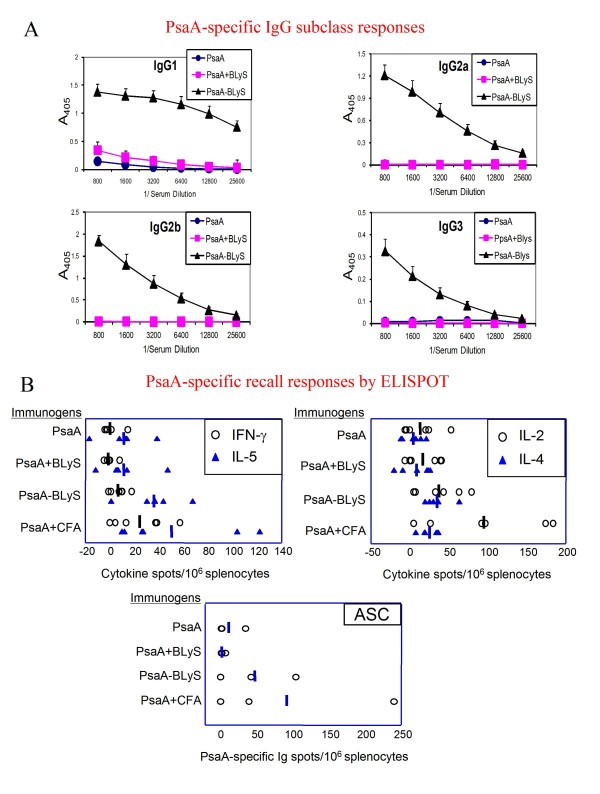
**Evaluation of antigen-specific immunoglobulin subclass and cytokine production**. A, The IgG subclass distribution of PsaA-specific antibodies was determined by ELISA for sera obtained from BALB/c mice at week 5 (following 2 immunizations as outlined in Figure 2). Data are presented as means ± SEM for 5 mice per group. B, For ELISPOT assays, C3H/HeJ mice immunized with the indicated antigens at week 0, and boosted with PsaA alone at week 2. Splenocytes were prepared from individual mice at week 3 were stimulated with soluble PsaA for the evaluation of PsaA-stimulated cytokine secreting cells, or with ELISPOT plate-immobilized PsaA for the enumeration of PsaA-specific antibody secreting cells (ASC). The data for cytokine spots are combined from two experiments with 3 mice per group and the data for ASC are from one experiment with 3 mice per group. Spots from individual mice and group means are shown.

Evaluation of the IgG subclass distribution of PsaA specific antibodies elicited by the different PsaA immunization regiments are summarized in Figure 3A. These data demonstrated that mice immunized twice with PsaA, or PsaA + BLyS exhibited PsaA specific antibodies exclusively of the IgG1 subclass. These results contrasted markedly with the subclass distribution of PsaA-BLyS immunized mice, which exhibited dramatic elevations for IgG1, IgG2a, IgG2b and IgG3 subclasses. Contrary to our expectations however, none of the immunization regimens resulted in a PsaA-specific serum IgA response (data not shown). Collectively these data indicate that covalent linkage of BLyS to a test antigen results in a highly immunogenic protein, which is more effective at eliciting antibody responses than is the test antigen merely admixed with BLyS.

### Evaluation of PsaA-specific cellular recall responses

The marked elevations in PsaA-specific serum IgG2a and IgG3 antibodies following immunization with PsaA-BLyS led us to become interested in evaluating, by ELISPOT, the cytokine secretion profiles of T cells generated by exposure to BLyS at the time of initial (primary) antigenic exposure. The data for cytokine secretion assays are summarized in Figure 3B. Spleen cells from mice primed with PsaA-BLyS responded with the production of IL-2, IL-4, and IL-5 and very modest but consistent IFN-γ production. Mice primed with PsaA or PsaA + BLyS exhibited only modest elevations in IL-2, IL-4 and IL-5 in response to PsaA stimulation, while the IFN-γ responses were below those observed for unstimulated control cultures. We used ELISPOT to evaluate frequencies of PsaA-specific antibody secreting cells in the spleens of immunized mice. PsaA-BLyS primed mice had larger numbers of PsaA-specific ASC than mice primed with either PsaA or PsaA + BLyS. Overall, evaluation of the cellular responses supports the conclusion that fusion of PsaA to BLyS resulted in a superior immunogen compared to PsaA mixed with (but not covalently linked to) BLyS, or to PsaA alone.

### Evaluation of antibody responses in TACI-deficient mice

**Figure 4:**
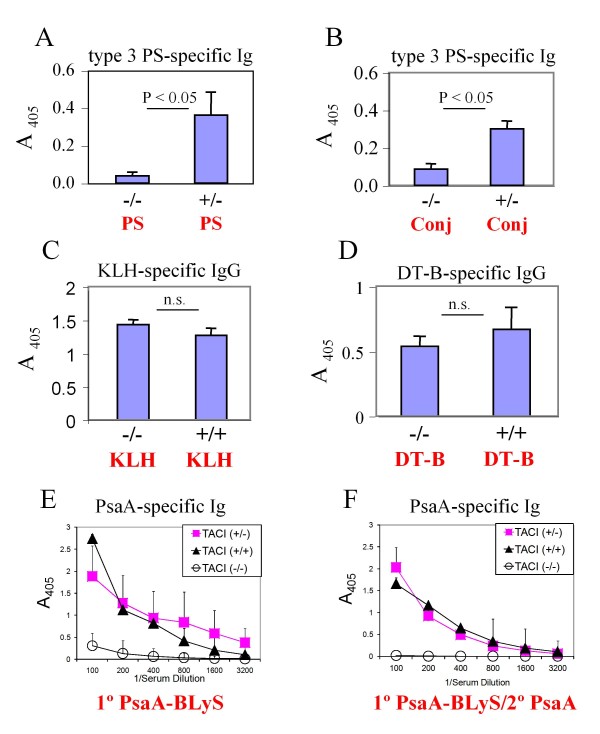
**Evaluation of antigen-specific immunoglobulin responses in TACI-deficient mice**. TACI-deficient mice (-/-), wild type (+/+) or heterozygous (+/-) littermates were immunized with type 3 PS [PS] (A) or type 3 PS-CRM_197 _conjugate [Conj] (B) and evaluated for PS specific serum antibodies by ELISA 2 weeks later. TACI-deficient or wild type littermates were immunized at weeks 0 and 2 with KLH [50 μg/mouse] (C), or DT-B [2 μg/mouse] (D), and evaluated for specific antibody at week 4 by ELISA. For PsaA-specific serum antibody responses, TACI-deficient, wild type, and heterozygous mice were immunized with 100 pmol of PsaA-BLyS fusion protein at week 0 (1°) and boosted with PsaA at week 3 (2°). Mice were bled at weeks 2 and 6 for the evaluation of primary (E) or secondary (F) PsaA-specific serum antibodies, respectively, by ELISA. Data are presented as means ± SEM for sera from individual mice (4-5 mice per group). In A-D A_405 _values for preimmune sera (diluted 1:100) were subtracted to generate specific absorbance for sera at 1:100 dilution.

A previous report demonstrated that mice deficient in expression of TACI, one of the 3 known BLyS receptors, are unable to mount normal antibody responses to polysaccharide antigens (TI-2), while the antibody responses to T-dependent (TD) protein antigens remain normal [22]. We immunized mice with a variety of antigens to characterize the antibody responses. Our data demonstrate that TACI-deficient mice mount an attenuated primary PS-specific antibody response following immunization with either purified type 3 PS (Figure 4A) or to a type 3 PS-CRM_197 _conjugate vaccine (Figure 4B). TACI-deficient mice were able to mount normal antibody responses to the TD antigens KLH (Figure 4C) and DT-B (Figure 4D). The primary serum antibody responses were also essentially identical between TACI-deficient and TACI-expressing littermates following a single immunization with either KLH or DT-B (data not shown). Immunization of mice with PsaA-BLyS, followed by a boost with PsaA alone revealed that TACI-deficient mice failed to mount a robust PsaA-specific serum antibody response following either the primary immunization (Figure 4E) or a boost with PsaA (Figure 4F). These results were interesting given our expectation that PsaA-BLyS would be acting as a TD antigen. In subsequent experiments, we demonstrated that immunization of TACI-deficient mice with PsaA in CFA followed by a boost with PsaA alone elicited PsaA-specific antibody responses of equal magnitude to those of TACI-sufficient wild type (TACI ^+/+^) or heterozygous (TACI ^+/-^) mice (data not shown). These results support our contention that that the defective antibody responses to PsaA-BLyS by TACI-deficient mice point to a specific requirement for TACI in the immune response to PsaA-BLyS.

## Discussion

BLyS has been the subject of extensive investigations following its initial discovery and characterization as a B lymphocyte stimulator. Many of the investigations have focused on characterizing the role of BLyS in the generation and maintenance of B cell driven autoimmune conditions such as systemic lupus erythematosus, and rheumatoid arthritis. We undertook these experiments to characterize the effects of BLyS on the induction of cellular and humoral immune responses to a heterologous antigen. Our experiments suggest that BLyS is a potent molecular adjuvant when administered to mice in the form of a fusion protein conjugated to the target antigen.

A mild adjuvant effect of BLyS was observed for mice immunized with admixtures of BLyS and PsaA, and moderate increases in PsaA-specific antibodies were only evident following two immunizations with the admixture. These PsaA-specific antibody responses were further increased in the tertiary response following a boost with PsaA alone. This enhancing effect of BLyS coadministration on PsaA-specific antibody responses is consistent with previous reports demonstrating the ability of BLyS to enhance the viability of suboptimally activated B cells [23], and to promote the survival of plasmablasts [24]. We therefore suggest that enhanced viability of PsaA-specific B cells/plasmablasts, rather than other mechanisms, such as increased T cell help was responsible for the observed difference in antibody responses between PsaA + BLyS and PsaA. This notion is indirectly supported by our ready demonstration of a survival effect of BLyS on cell viability *in vitro*, and because the PsaA-specific cytokine production elicited by primary immunizations with PsaA + BLyS and PsaA alone were similar. Although the tertiary antibody responses following two immunizations with PsaA + BLyS admixtures followed by PsaA alone were almost as high as the tertiary antibody responses observed for two immunizations with the PsaA-BLyS fusion protein followed by PsaA alone, this response remained restricted to the IgG1 subclass in the former cohort, while the latter cohort maintained the broad IgG subclass distribution observed for the secondary responses to PsaA-BLyS (data not shown). Conjugation of BLyS to PsaA clearly produced increased PsaA-specific responses over those observed with PsaA and BLyS admixtures. We hypothesize that this resulted from efficient antigen capture via BLyS specific receptors and subsequently improved antigen presentation and T cell costimulation, as has been shown for a model system utilizing a fusion of antigen to IL-2 [25]. The broad IgG subclass distribution of IgG responses elicited in response to immunization with the fusion protein suggest that the modest increases observed in antigen specific IFN-γ production may have been sufficient to drive the production PsaA-specific IgG2a [26]. Alternatively, the efficient production of this subclass and other IgG subclasses may have been driven by BLyS mediated enhancement of cytokines not studied here, such as IL-27 which has been shown to direct the production of IgG2a in the absence of IFN-γ [27].

Our results demonstrate that continuous administration of large amounts of BLyS are not required to observe at least some enhancing effects on the immune response to coadministered antigens, and suggests that unconjugated BLyS may find utility as an adjuvant administered with antigen as an admixed bolus, particularly in situations where conjugation to target antigens of interest is not feasible.

Our results demonstrate that the human ortholog of BLyS (~85% amino acid identity and 94% similarity to murine BLyS within the C-terminal extracellular domain) [1] is biologically active in mice. We observed a modest but reproducible anti-hBLyS response in mice immunized twice with PsaA-BLyS, but we did not investigate whether these antibodies were capable of inhibiting the biological activities of exogenous (human) or endogenous (murine) BLyS. Future studies to address the functional consequences of an anti-BLyS response are of interest [28], wherein we would evaluate the ability of multiple immunizations with PsaA-BLyS to attenuate disease progression and autoantibody production in an MRL/lpr mouse model of SLE or in a collagen induced arthritis mouse model of RA [29,30]. Strategies aimed at attenuating the biological activity of BLyS are justified, given the presence of increased levels of endogenous BLyS in the circulation of MRL/lpr mice [11], as well as the frequently observed elevation of BLyS in RA and SLE patients [5,6,31].

Numerous studies have demonstrated B cell and T cell co-stimulatory attributes of BLyS. Of the three receptors described for BLyS, BAFF-R has been identified as the main receptor on B and T cells through which BLyS exerts its co-stimulatory effects [19,32], while TACI has been shown to be dispensable for the proliferation of purified B cells in response to stimulation with BLyS and anti-μ [33]. The majority of B cells, as well as a subset of T cells express BAFF-R [19], while TACI is expressed on a subset of B cells, including marginal zone (MZ) B cells [19] and memory B cells [34]. Other studies have shown that splenic B cells, which normally express low levels of surface TACI can be induced to express high levels of TACI by stimulation with anti-μ [35] or toll-like receptor (TLR) ligands like CpG containing oligodeoxynucleotides (CpG ODN) [36]. Investigation of the expression of TACI on T cells has produced conflicting results. One report has shown that a polyclonal affinity purified antibody raised against TACI binds to a subset of phorbol myristoyl acetate (PMA)-activated human T cells [37], while another study with TACI-specific monoclonal antibodies raised against murine and human TACI failed to show TACI expression on the surface of resting or activated murine or human T cells, respectively [19]. The third receptor for BLyS, BCMA appears to be expressed exclusively on spleen and bone marrow plasma cells [34]. The initial characterization of mice deficient in BCMA revealed no overt abnormalities in the lymphoid cell compartment, and these mice mounted normal antibody responses to all antigens [38]. However, a more recent study has demonstrated that BCMA-deficient mice exhibit a defect in the survival and maintenance of long-lived bone marrow plasma cells [39].

Mice deficient in BAFF-R have reduced numbers of B cells (~10% of BAFF-R intact littermates) and exhibit attenuated responses to TD antigens, but close to normal responses to TI-2 antigens [40,41]. The phenotype of BLyS deficient mice is even more severe than that of BAFF-R deficient mice, and these mice exhibit impaired immunoglobulin responses to both TI -2 and TD antigens, in addition to ~10 fold reductions in total B cells and baseline immunoglobulin concentrations, relative to BLyS intact littermates [41,42]. By contrast, TACI-deficient mice have increased numbers of B cells (~200% of TACI intact littermates) and exhibit a defective response to TI-2 antigens, while retaining the capacity to mount normal responses to TD antigens [22,33]. On the basis of these published observations, we might have expected that BAFF-R would be the main receptor through which BLyS (as a biologically active fusion partner to the T dependent antigen PsaA) would exert its immunostimulatory effects. Our observation that the relatively potent immunogenicity exhibited by PsaA-BLyS depended on TACI expression was therefore unexpected. This observation suggests that the enhanced immunogenicity of PsaA-BLyS was not simply the result of a carrier effect wherein potentially immunogenic peptide fragments from human BLyS that are not identical to those derived from endogenous (murine) BLyS could provide additional linked T-cell-epitopes that could have contributed to the immunogenicity of the fusion protein. A possible explanation for our results is provided by recent article that has reported very high levels of TACI expression on murine bone marrow-derived dendritic cells (BMDC) [30]. Expression of BAFF-R and BCMA was also observed on these BMDC preparations, but at much lower levels than observed for TACI. A separate study has also reported TACI expression, but no BAFF-R expression on human monocyte-derived dendritic cells (DC) [43]. The discovery of TACI expression on DC is key, because DC are widely acknowledged to be essential to the priming of naïve T cells for a productive immune response *in vivo *[44]. If the significant expression of TACI on DC is confirmed for naturally arising DC populations in the mammalian host, it would suggest that the potent immunogenicity of PsaA-BLyS is primarily facilitated by the binding of the fusion protein to DC via TACI. In the absence of evidence for TACI expression by DC, we would be prompted to entertain the hypothesis that B cells were the primary APC (by virtue of their expression of TACI) responsible for the enhanced immunogenicity of PsaA-BLyS. Although there is evidence that antigen-specific B cells can serve as effective APC for naïve CD4^+ ^T cells specific for the same antigen [45], the large number of such antigen-specific B cells that would be required *in vivo *to have an impact makes this hypothesis less appealing.

The observation that T cells derived from a TACI-deficient mouse strain (independently generated on a genetic background similar to that used for the TACI-deficient mice used in our experiments) respond to *in vitro *co-stimulation with anti-CD3 and BLyS in a fashion similar to wild type (TACI sufficient) littermates [19] suggests that a deficit in T cell responses due to TACI ablation cannot explain the attenuated immunogenicity of PsaA-BLyS in our TACI-deficient mice. It is also curious that the expected presence of PsaA-specific activated T cells apparently failed to support a PsaA-specific antibody response via other co-stimulatory pathways known to be critical for T-dependent (TD) antibody responses, such as the CD40-CD40L (CD154) axis [46].

In summary, our data are consistent with the hypothesis that DC-mediated antigen presentation of PsaA-BLyS via the binding of BLyS to TACI is essential for initiating (priming) the PsaA-specific immune response, followed by subsequent enhancement of this immune response via BLyS mediated agonistic effects such as immunoglobulin class switching [47], enhanced B and plasma cell survival [14], and T cell co-stimulation [19,32].

Future studies using mice deficient in the other two known receptors for BLyS (BAFF-R and BCMA) should help to define the relative importance of each BLyS receptor in the induction of immune responses by PsaA-BLyS. Additionally, immunization of CD4^+ ^T cell-deficient mice [48], and mice deficient in CD40L [49] (which have defective antibody responses to TD antigens but intact responses to TI antigens) with the PsaA-BLyS fusion may shed more light on the T-dependence or -independence of the response to PsaA-BLyS.

One of the main arguments against using BLyS as an adjuvant is its propensity to promote autoantibody production when present at elevated concentrations for prolonged periods of time. The fact that we were unable to demonstrate an elevation in dsDNA specific antibodies following one or two immunizations with a BLyS containing protein antigen would argue that transient elevation in BLyS concentrations by itself might not be sufficient to induce autoantibody production. Another possibility is that the seemingly modest BLyS-specific antibody response elicited by our constructs might have been capable of neutralizing some of the biological activities of BLyS and thereby attenuating autoantibody production. Alternatively, the amounts of BLyS administered in our experiments may have been insufficient to drive self-sustaining autoantibody production. Consistent with the latter view is the observation that while daily injections of mice with up to 75μg of BLyS (3 mg/kg) for 14 days resulted in elevated quantities of total serum antibodies, these antibody levels returned to baseline within 2 weeks following cessation of the BLyS injections [13].

The utility of BLyS as a vaccine adjuvant has recently been demonstrated in a murine model of pulmonary *Pseudomonas aeruginosa *infection [50]. In this study mice administered an adenoviral vector containing BLyS coding sequences (adjuvant) along with a heat killed whole cell *P. aeruginosa *vaccine (PA) exhibited higher antibody concentrations than mice vaccinated with either PA alone or PA and an empty adenoviral vector. BLyS co-administration elicited elevated numbers of PA-specific CD4^+ ^T cells secreting IFN-γ but not IL-4. The BLyS-treated PA-vaccinated mice were solidly protected from lethal pulmonary challenge with live *P. aeruginosa*, while PA vaccinated mice not receiving BLyS were not significantly protected. This protection correlated positively with a substantial reduction of live bacteria in the lungs of challenged mice, and was dependent on CD4^+ ^T cells, as vaccination of mice deficient in CD4^+ ^T cells with both PA and BLyS were unprotected. The critical contribution of CD4^+ ^T cells to protection was further underscored by the observation that BLyS co-administration elicited similar PA-specific antibodies in CD4^+ ^T cell intact and CD4^+ ^T cell-deficient mice.

## Conclusions

There is need for more and improved vaccines that protect against pneumonias with a bacterial aetiology. Although antibody-dependent mechanisms have long been assumed to be exclusively responsible for protection against extracellular bacteria at mucosal surfaces, several reports have recently demonstrated an antibody-independent role for CD4^+ ^T cells in protective immunity against respiratory infections. To that end, IL-17 [51] and IFN-γ [52] production by CD4^+ ^T cells have been shown to be protective. The protective action of both IL-17 and IFN-γ results from the ability of these cytokines to direct the activation and infiltration of neutrophils to sites of infection for enhanced bacterial killing. As such, the development of protective vaccines against mucosal infection must incorporate the capacity for eliciting cell-mediated (CD4^+ ^T cell) responses characterized by IFN-γ and/or IL-17 production in response to antigens from such pathogens.

This study suggests that covalent attachment of BLyS can be an effective adjuvant strategy that might yield improved vaccines. The elicitation of PsaA-specific antibodies belonging to the IgG1, IgG2 and IgG3 subclasses, as well as the modestly increased numbers of IFN-γ secreting cells supports the notion that BLyS effectively facilitates the induction of a broad range of humoral and cell-mediated immune responses. Another study has also demonstrated that BLyS dramatically expands IL-17 secreting CD4^+ ^T cells [30]. Collectively, these data suggest that the effectiveness of vaccines that protect against bacterial pneumonias caused by gram-positive (*Streptococcus pneumoniae*) and gram-negative (*P. aeruginosa*) organisms can be enhanced by exploiting the immunostimulatory attributes of BLyS as an adjuvant.

## Methods

### Mice and immunizations

Six- to eight-week old female BALB/c, C3H/HeJ, and MRL/MpJ-*Fas*^*lpr*^/J (MRL/lpr) mice were purchased from The Jackson Laboratory, Bar Harbor, Maine. Mice deficient in TACI have been previously described [22]. The genetic background of the TACI knockout mouse colony is equally 129SVJ and C57Bl/6 [22], and brother sister matings were used to generate TACI^+/+^, TACI^+/- ^and TACI^-/- ^genotypes. The genotyping of TACI mice was performed by PCR using the primers MTS-1, 5' CCTCAGGCCAGGAGCTTTTAGGGAGAA 3'; MT-AS2: 5' CCAGCATCCCCTCTGCTCTGGTTTTAT 3'; and NEO-S1, 5' CCTGGGTGGAGAGGCTTTTTGCTTCCT 3'. Primers MT-S1 and MTAS2 amplify a 440 bp PCR fragment from the wild type locus and primers Neo-S1 and MT-AS2 amplify a 300 bp PCR fragment from the disrupted TACI locus. The PCR conditions used were one cycle at 95°C for 5 minutes, followed by 35 cycles (92°C for 1 min., 64°C for 1 min., and 72°C for 1 min.) followed by a 10 min. cycle at 72°C. Amplified products were electrophoresed through 1.2% Tris-borate-EDTA buffered agarose gels, and stained with ethidium bromide to visualize the amplified PCR products.

All mice were housed under specific pathogen-free conditions, with sterile food and water *ad libitum*. The Case Western Reserve University Institutional Animal Care and Use Committee approved all animal experiments.

For immunizations, groups of 5-6 mice were injected intraperitoneally (i.p.) with antigens in 100 μl of phosphate buffered saline (PBS). Mice were immunized with 100 pmol of recombinant antigens (PsaA, PsaA-BLyS, BLyS, or equimolar mixtures of BLyS and PsaA) unless otherwise indicated. In some experiments synthetic phosphorothioate CpG containing oligodeoxynucleotide (ODN) 1826 (5' TCCATGACGTTCCTGACGTT 3') and non-CpG ODN 1972 (5' TCCAGGACTTCTCTCAGGTT 3') (gifts from Clifford Harding, Case Western Reserve University) were used as adjuvant and control ODNs, respectively at 50 μg admixed with 100 pmol PsaA in PBS per mouse. Complete Freund's Adjuvant (CFA; Sigma) was used as a 1:1 (v/v) emulsion with 100 pmol PsaA in PBS. Sera were prepared from blood collected from mice via the tail vein and were stored at ^-^20°C until used for assays.

### Plasmid construction and recombinant protein purification

A plasmid encoding the soluble extra-cellular domain of human BLyS was constructed by cloning a PCR amplified fragment of BLyS (amino acids 140-285) into pET27b+ (Novagen) at the *Nco*I and *Xho*I sites. A plasmid encoding PsaA fused to the N-Terminus of soluble BLyS was made by ligating the PsaA coding sequence from the previously described p29-psaA plasmid [17] into pET27-BLyS at the *Xba*I and *Hind*III sites. This action replaced the signal peptide sequence in pET27-BLyS with an S-Tag sequence. Recombinant proteins were expressed and purified from lysates of the *E. coli *expression strain BL21(DE3) (Novagen) by metal affinity chromatography according to the manufacturers instructions (Novagen). The purified recombinant proteins were dialyzed extensively against PBS containing 50 mM Tris (pH 8.8). Protein concentrations were estimated using a Bradford kit (Bio-Rad) and filter sterilized using 0.22 μm syringe filters (Millipore) prior to storage at 4°C. Endotoxin (LPS) contamination of recombinant proteins was measured using a limulus amebocyte lysate kit (LAL; Sigma) and were determined to be below the level of detection (<50 Endotoxin units/ml). Additional recombinant proteins used in these experiments were non-lipidated putative protease maturation protein A (PpmA; amino acids 21-309) of *Streptococcus pneumoniae *[53], and the non-toxic B fragment of diphtheria toxin (DT-B, amino acids 202-535) [54]. Each was cloned as a PCR amplified gene fragment into pET29b+ plasmid (Novagen) at *Nco*I and *Xho*I. These recombinant proteins were expressed and purified from lysates of recombinant *E. coli *as described above. Additionally, keyhole limpet hemocyanin (KLH) purchased from Sigma was used as a T-dependent antigen, and type 3 capsular polysaccharide (PS) from *Streptococcus pneumoniae *was used as a Type 2 T-independent antigen. A glycoconjugate vaccine consisting of Type 3 PS covalently linked to diphtheria toxoid (CRM_197_) was a gift of Dr. Ron Eby, Wyeth Vaccines.

### Gel electrophoresis and Western blot for detection of recombinant proteins

Recombinant proteins were subjected to sodium dodecyl sulfate-polyacrylamide gel electrophoresis (SDS-PAGE) under reducing conditions, and either stained directly with Coomassie blue to visualize the proteins, or else electrophoretically transferred to polyvinylidene difluoride (PVDF) membranes (Bio-Rad, Hercules, CA) for protein detection by Western blot. The PVDF membranes were reacted first with polyclonal mouse PsaA-specific antisera, followed by incubation with alkaline phosphatase conjugated goat anti-mouse IgG. Positively reacting protein bands were visualized by incubation in 5-bromo-4-chloro-3-indolylphosphate-nitroblue tetrazolium (BCIP-NBT) chromogenic phosphatase substrate (Sigma).

### *In vitro *assay for BLyS bioactivity

Standard co-stimulation assays were performed to demonstrate that the recombinant BLyS proteins produced for these experiments retained their ability to stimulate B cells. Splenocytes from C3H/HeJ mice or BALB/c mice were seeded into individual wells of a 96 well plate at 5 × 10^5 ^cells per well in RPMI supplemented with 10% fetal bovine serum (RPMI-10). Serial dilutions of recombinant PsaA or PsaA-BLyS in RPMI-10 and a F(ab')_2 _fragment of goat anti-mouse IgM (μ heavy chain specific) in RPMI-10 were added to wells in triplicate. The final volume was 200 μl per well. Plates were incubated for 48 hours at 37°C in 5% CO_2_. Alamar blue (Accumed; 20 μl per well) was added at 48 hrs, and plates were incubated at 37°C for an additional 20-24 hours. Absorbances at 570 nm and 600 nm were read using a Bio-Rad plate reader. Data are presented as specific absorbance (A_570_-A_600_). Increased absorbance is directly proportional to the amount of reduction of Alamar blue in stimulated cultures, which in turn is directly proportional to the number of viable cells in each well [55]. Recombinant PsaA and PsaA-BLyS were tested at 1000 nM - 15.6 nM in four-fold dilution steps, and F(ab')_2 _anti-μ or control goat F(ab')_2 _were each used at 1 μg/ml. Similar T cell co-stimulation assays were performed using recombinant BLyS and a hamster anti-mouse CD3ε monoclonal antibody (Southern Biotechnology Associates, Birmingham, AL) as the primary T cell stimulus. An isotype-matched hamster antibody of irrelevant specificity from the same vendor was used as a control in these assays. The Alamar blue procedure described above was used for the evaluation of T cell proliferation by splenocytes from BALB/c and C3H/HeJ mice.

### Characterization of antibody responses

ELISA was used to characterize PsaA-specific antibody responses in mice immunized with PsaA, PsaA and BLyS or PsaA-BLyS fusion protein generated in these experiments. Briefly, 96-well plates **(**Immulon I, Dynatech, Chantilly, VA) were coated with recombinant PsaA (2 μg/ml, 100 μl per well) overnight at 4°C. Serial dilutions of sera were added to the wells in duplicate. Alkaline phosphatase (AP)-conjugated antibodies specific for murine immunoglobulins were used as the secondary reagent. The plates were developed by adding *p*-nitrophenyl phosphate (Sigma) and read at 405 nm using a spectrophotometer (Molecular Devices, Inc.). Relative antibody concentrations were determined by performing ELISA on serial dilutions of individual sera. AP-conjugated antibodies specific for murine immunoglobulins (Ig) were purchased from Southern Biotechnology Associates, Birmingham, AL.

To test for the elicitation of human BLyS-specific antibodies following immunization of mice with the BLyS-containing recombinant proteins and mixtures described above, ELISA assays were performed as described above, except that the microtiter plates were coated with recombinant BLyS (2 μg/ml, 100 μl/well).

To test for the elicitation of autoantibodies in mice that had received BLyS or PsaA-BLyS as part of the immunization regiment, we performed ELISA assays to detect the presence of double-stranded (ds) DNA specific antibodies. Immunlon I plates were coated with calf thymus DNA (Sigma) at 2.5 μg/ml, 100 μl per well overnight at 4°C. Plates were washed and incubated with serial dilutions of antisera from immunized mice. Sera from 6-month-old MRL/MpJ-*Fas*^*lpr*^/J (MRL/lpr) mice were included as positive (dsDNA reactive) controls, and sera from 3-month-old BALB/c mice were included as negative (dsDNA non-reactive) controls. Following washes, bound antibodies were detected using AP-conjugated goat anti-mouse Igκ, after incubation with *p*-nitrophenyl phosphate as described above.

Antibodies specific for KLH and DT-B were assessed by ELISA in the sera of KLH and DT-B immunized mice using Immulon 1 microtiter plates coated with KLH (10 μg/ml, 100 μl per well) or DT-B (2 μg/ml, 100 μl per well), and were developed as described for PsaA-specific antibody detection. Type 3 PS-specific antibodies elicited in response to immunization with type 3 PS or its CRM_197 _conjugate were assessed by ELISA using Polysorp plates (Nunc, Roskilde, Denmark) coated with type 3 PS (10 μg/ml, 100 μl/well), as previously described [17].

### Evaluation of cellular PsaA-specific recall responses

Cytokine recall responses specific for PsaA were evaluated using an enzyme linked immunospot (ELISPOT) assay. This assay has been shown to be suitable for enumeration of low frequencies of antigen specific cellular responses following *in vivo *priming with antigen [56]. Assays were performed essentially as described, with minor modifications [56]. Briefly, plates (ImmunoSpot, Resolution Technology, Columbus, OH) were coated overnight at 4°C with the cytokine-specific capture Abs specified below. The plates were blocked with 1% BSA in PBS for 1 h at room temperature and washed four times with PBS. Subsequently, freshly isolated spleen cells from groups of mice primed with PsaA, PsaA + BLyS, PsaA-BLyS fusion protein, or PsaA in complete Freund's adjuvant, and boosted with PsaA in PBS were plated at 1 × 10^6 ^per well in flat-bottom, 96-well microtiter plates in serum-free HL-1 medium (BioWhittaker, Walkersville, MD) supplemented with L-glutamine at 1 mM, in the presence or absence of recombinant PsaA. After 24 or 48 h of cell culture in the incubator, the cells were removed by washing three times with PBS and four times with PBS containing 0.05% Tween (PBST). Detection Abs were added and incubated at 4°C overnight (either horse radish peroxidase (HRP)-labeled or biotinylated). The plates were washed three times with PBST. For biotinylated detection mAbs, streptavidin-HRP (Dako, Carpenteria, CA) was added at 1:3000 dilution, incubated for 2 h at room temperature, and removed by washing twice with PBST and twice with PBS. The spots were visualized by adding HRP substrate 3-amino-9-ethylcarbozole (Pierce, Rockford, IL). The following combinations of capture and detection mAbs were used for IL-2, IL-4, IL-5, and IFN-γ assays respectively: JES6-1A12 (5 μg/ml) and JES6-5H4-biotin (2 μg/ml), BVD4-1D11 (2 μg/ml) and BVD4-24G2-biotin (2.5 μg/ml), TRFK5 (5 μg/ml) and TRFK4-HRP (2 μg/ml), and R46A2 (5 μg/ml) and XMG1.2-HRP (2 μg/ml). Image analysis of ELISA spot assays was performed on a Series 1 ImmunoSpot Image Analyzer (Resolution Technology, Columbus, OH) customized for analyzing ELISA spots to meet objective criteria for size, chromatic density, shape, and color. Detection of PsaA-specific antibody secreting cells (ASC) was assessed for splenocytes from immunized mice by a slight modification of the ELISPOT assay described above. Briefly, plates were coated with PsaA in place of cytokine capture antibodies, and splenocytes were added to wells, as above. Following incubation, detection of total PsaA-specific ASC was effected by incubation of washed wells with HRP-conjugated anti-mouse Igκ, followed by spot development with 3-amino-9-ethylcarbozole.

## Reviewers' comments

### Reviewer 1: Jonathan Yewdell, National Institute of Allergy and Infectious Diseases, Laboratory of Viral Diseases

Immunogenicity, the ability of a substance to elicit an immune response, is the difference between a vaccine protecting an individual against a given infectious disease *vs*. the individual suffering the consequences of infection. There are two elements to immunogenicity. One is the antigen itself. The antigen must be given in a form that elicits the right type of adaptive immune response, which is some combination of antibodies (produced by B cells) and T cells. The other element is the adjuvant, which activates elements of the innate immune system to in turn license B and T cells to become activated.

The need for adjuvants (Charlie Janeway's famous Dirty Little Secret (1)) to induce effective immunity to most non-infectious antigens (infectious agents typically have considerable adjuvant activity of their own) is probably the greatest stumbling block to improving vaccines to infectious diseases. A significant number of individuals simply don't respond to many vaccines, a problem that is greatly exacerbated with age. Better adjuvants would also allow use of lower doses of antigen, saving money and potentially lives, when to cite a cogent example, amounts of vaccine are limited and Swine flu is on the march.

In the past, adjuvants were based on empirical observation of increased immunogenicity. Many of the best adjuvants (*e.g*. complete Freund's adjuvant, heat killed mycobacteria emulsified in mineral oil) have such severe side effects that their use is proscripted even in experimental animal studies. There is but one FDA approved adjuvant for use in humans, alum (alas).

After 30 years of basic research into the molecular basis of immunity, the fruits are beginning to ripen, and there is a wealth of information regarding the molecular switches downstream of adjuvant activation of innate immunity. Gor *et al*. focus on one such switch, the wonderfully named BLyS (B lymphocyte stimulator), expressed by immune cells to interact with receptors on B and T lymphocytes. They convincingly show that conjugation of BLyS synthesized in bacteria to a medically important antigen (pneumococcal surface adhesin A) increases antibody responses, in a manner dependent on one of the three known BLyS receptors, TACI. Intriguingly, the adjuvant effect of BLyS required covalent conjugation of BLyS to antigen (i.e. immunizing a mixture of antigen plus BLyS did not enhance immunogenicity), pointing the way to future studies on the processing and presentation of antigen to B cells, a field with tremendous opportunity for making basic discoveries, not to mention additional studies on other antigens (e.g. flu vaccine).

In summary, this is a fine study in an area of research, immune adjuvant development, of great interest from both basic and translational aspects. One of the most telling findings is Figure 2, panel H, where the impressive adjuvant effects of BLyS still fall far short of complete Freund's adjuvant, pointing to the need for increased funding for adjuvant research.

1. Approaching the asymptote? Evolution and revolution in immunology. Janeway, C.A.Jr. Cold Spring Harb. Symp. Quant. Biol. 54, 1-13 (1989).

#### Author's response

We thank the reviewer for his comments.

### Reviewer 2: Michael Cancro, Department of Pathology and Laboratory Medicine, University of Pennsylvania School of Medicine (nominated by Andy Caton, Wistar Institute)

The fusion protein is expressed as expected and the fused BLyS retains biological activity, based on the viability assays in Figure 1.

The data in Figure 2 show that the fusion protein yields higher titres of PSA specific antibodies than immunization with PSA alone or PSA with simultaneously administered free BLyS. These data also indicate that no dsDNA-binding antibodies are generated upon a brief BLyS or PSA-BLyS exposure. Accordingly, the central idea may have merit, but questions remain regarding the basis for and import of these enhanced titers.

First, it is not clear whether this reflects the biological activity of BLyS-BLyS receptor interactions in promoting B cell survival/activation per se, or instead represents a "carrier" effect that enhances T cell activation, and does not rely on BLyS's native biological activity. The fusion protein involved human BLyS with some linkers, and will thus contain sequences that might be presented to and recognized by mouse T cells, making this a plausible possibility. Since a carrier effect requires linked recognition, treatment groups with simultaneous free BLyS administration do not address this concern. A number of approaches might be taken to address this, but the most incisive might be a direct measure of whether any T cells specific for human BLyS epitopes are generated. This might be accomplished by a T cell proliferation or IL-2 induction assay using APCs pulsed with hBLyS plus T cells from the PSA-hBLyS primed mice. Alternatively or in addition, one might construct similar fusion proteins with either human BLyS that has had its receptor-binding site disrupted, which should not enhance; or with mouse BLyS, which should.

The second question arises upon noting that antibody levels are enhanced about 5-fold over controls in most of the primary immunizations. This increase is fairly modest, and is dwarfed by the ~1000-fold differences observed when CFA is used as an adjuvant. Thus, one wonders what the absolute magnitude (on a weight per volume basis) of the antibodies might be, and whether the increases in titre would afford increased protection. Accordingly, the paper would be strengthened by a fully quantitative (mcg/ml) assessment of the serum antibody, and by including some measure of whether the increases with BLyS-PSA yield significant shifts in the infectivity per se or in an in vitro surrogate for virulence/adhesion.

The results with the TACI deficient mice are intriguing, although they seem somewhat tangential to the rest of the manuscript. While the observation is interesting, additional experiments are required to assess their import and relationship, if any, to the main thrust of the article.

A minor point is the implication that the viability assay used in Figure 1 necessarily reflects cell division. This is unlikely in panel D of Figure 1, inasmuch as BLyS alone does not induce cell division, but indeed fosters survival in vitro. Assays directly discerning survival and division, rather than just the relative number of viable cells at the end of culture, would more directly interrogate the biological activities of the fusion product (e.g.; Flow cytometric analysis of CFSE plotted against either TOPRO or DAPI). Nonetheless, the biological activity of BLyS in the fusion protein indeed seems to be preserved. This point is only made for precision.

#### Author's response

We did consider the possibility, cited by the reviewer, that the enhanced immune response to PsaA covalently linked (versus not linked) to BLyS could have been due to carrier function as opposed signalling through BLyS receptors. While we agree that mutating the receptor-binding site on BLyS would have been a valuable way to address the importance of signalling through BLyS receptors, when these studies were carried out, the receptor-binding site on BLyS was not defined.

In support of the interpretation that the increased immunogenicity of PsaA-BLyS versus PsaA alone or PsaA + BLyS, TACI-deficient mice exhibited a smaller increment than TACI-expressing mice in the antibody response as a result of covalently linking BLyS to PsaA. Since earlier work (von Bülow et al., 2001) and our own studies established that TACI-deficient mice responded normally to other typical protein (i.e., thymus-dependent, or TD) antigens, we think it is unlikely that the substantially decreased magnitude of the antibody response to PsaA-BLyS of TACI -/- mice, in comparison to TACI +/+ mice, is attributable to an effect of TACI-deficiency on the T cell response. However, we acknowledge that it would have been useful to assess the CD4^+ ^T-cell response to human BLyS in the mice immunized with PsaA-BLyS.

We agree that quantitation of antibody responses in mcg/ml is preferable when possible. When these studies were performed there were few commercial reagents available, and we did not have a monoclonal antibody specific for BLyS to use in establishing the standard curve required for such quantitation.

The results with the TACI-deficient mice are not tangential in our view. Since as noted, above, TACI-deficient mice responded normally to other typical protein (i.e., thymus-dependent, or TD) antigens (KLH and diphtheria toxoid; also see von Bülow *et al*., 2001), we believed it was relevant that TACI -/- mice exhibited a reduced response to PsaA-BLyS, relative to TACI +/+ mice. This result argues against BLyS functioning purely as a carrier protein, since if TACI-deficiency substantially reduced the responses of CD4^+ ^T cells, then we and others (von Bulow *et al*., 2001) should not have found that TACI -/- mice generated antibody responses to various protein immunogens comparably to TACI +/+ mice. We do agree with the reviewer that there are other aspects of the role of TACI that would require further experiments.

The figure legend has been modified to reflect the reviewer's insight, which is appreciated.

### Reviewer 3: Rachel Gerstein, Department of Microbiology and Physiological Systems, University of Massachusetts Medical School

PsaA is a protein antigen from *Streptococcus pneumoniae*. Efforts to generate a potent vaccine for S.p. that protects independent of capsule antigen serotype have not succeeded. Since the serotype is determined by the capsular polysaccharide, investigation of protein antigens from *S.p*. is important. Also, a serious limitation for vaccines are the adjuvants available for use in humans; the most potent known adjuvants used in animal models cause side-effects, and so new adjuvants that are both effective and safe for use in humans are needed.

It would be helpful to state in more detail the rationale for using PsaA as a model antigen.

BLyS is an essential cytokine that controls B cell development and homeostasis. The authors made a fusion protein, PsaA-hBLyS, which is readily expressed in *E. coli *and purified. When cultured with mouse splenocytes, more cells are recovered when compared to culture with Spa alone or a sub-optimal amount of anti-μ to stimulate B cell proliferation. It is clear from the literature that BLyS promotes increased B cell viability and/or prevents apoptosis, so the experiment shows that the fusion has the activity expected for bio-active BLyS on mouse B cells.

PsaA-specific antibody responses in mice were determined using ELISA.

Importantly, anti-DNA abs are not detected (BLyS over-expression *in vivo *leads to lupus and other autoimmune manifestations). Particularly impressive are the levels of specific Ag when the secondary response is measured - much higher than when PsaA + BLyS is given as separate molecules in priming. Interestingly, PsaA alone can boost if PsaA-BlyS OR PsaA + BLyS is given in the primary. The authors discuss potential mechanisms behind this observation.

One goal of the study was to consider this fusion system as an adjuvant strategy - in this regard, when PsaA-BLyS was compared to PsaA+CFA, CFA was the most potent but PsaA-BLyS is better than PsaA+CpG (another adjuvant that can promote antibody formation), so the BLyS conjugate does have activity as an adjuvant.

In Figure 3, the broad IgG subclass distribution of the response to PsaA-BLyS is documented. Also in Figure 3, ASC are measured 1 week after boost (in spleen) and very few are detected. Although not central to the paper, the authors might consider using the same time points after boost as the ELISA data and also examining the bone marrow, a major repository of ASC.

In Figure 4, TACI-deficient mice are compared to WT and heterozygous deficient mice. Interestingly, when mice are immunized using PsaA-BLyS and boosted with PsaA, the TACI KO do not respond well when either primary or secondary responses are measured. The interpretation and discussion of this result are thought provoking, given that the major defect previously noted in TACI-deficient mice is in responses to TI-2 antigens, and PsaA is assumed to be a TD antigen. Given the documented role of BLyS in augmenting TI responses, it seems worth considering as to whether there is a TI-component to the PsaA-BLyS response.

Overall, the Ms reports data that will be of interest to immunologists and vaccinologists. The experiments are done with appropriate rigor and reveal a potent affect for boosting specific antibody responses without resorting to problematic strong adjuvants.

#### Author's response

We have added text to the Background section (p. 6) addressing the rationale for using PsaA as a model antigen.

The reviewer has made the excellent point that the effect of BLyS is increased cell viability and not proliferation. We have altered the figure legend to indicate that this is a viability/proliferation assay. In the Materials and Methods section ("***In vitro *assay for BLyS bioactivity")**, we have described the Alamar Blue assay which was used to assess increased cell numbers, whether due to proliferation (as in anti IgM treated cultures) or to increased viability (as in cultures containing recombinant BLyS).

We have added material to the Discussion section on the ability of PsaA alone to boost if PsaA-BlyS or PsaA + BLyS is given in the primary.

With reference to cytokines measured, we used an ELISPOT assay. The numbers of cytokine secreting spots are not very high using this assay, even for mice immunized with CFA, but they are clearly elevated for PsaA-BLyS compared to PsaA or PsaA + BLyS primed mice. In subsequent studies, we might increase the number of immunizations administered prior to assessment of cellular responses to accentuate any possible differences between groups that are reflected in the antibody production data.

We have changed the text in the abstract and throughout to reflect the very modest but consistent IFN-γ production observed.

The reviewer has also made very reasonable points about using matching time points for the cellular and humoral responses, as well as including an assessment of ASC in the bone marrow to obtain a more complete picture of the responses elicited, and we concur.

To address the insightful comments by the reviewer, we would like to briefly revisit some of our experimental observations and the assumptions underpinning our interpretation and presentation of the data.

We did not perform specific experiments to determine whether the PsaA-BLyS response is a T cell dependent (TD) response in the classical sense, in that we have not performed immunization experiments using CD4^+ ^T cell-deficient mice. Rather, we proceeded with the calculated assumption that PsaA is a TD antigen. This is supported by circumstantial observations such as the lack of repeating motifs in the primary amino acid sequence for PsaA, the monomeric nature of the recombinant protein (as determined by migration patterns through non reducing/non denaturing polyacrylamide gel electrophoresis), the non-immunogenicity of PsaA in the absence of adjuvants in both BALB/c and C3H/HeJ mouse strains, and the ability to elicit elevated secondary antibody responses dominated by the IgG subclasses other than IgG3, following primary immunization with standard adjuvants (CFA, IFA). These features are not characteristic of TI antigens.

Perhaps we have inadvertently converted it (PsaA) to a TI antigen by fusion to BLyS. Indeed one (we) should not be surprised if some aspects of the immune response resemble those elicited in response to classical T cell independent (TI) antigens, given that one of the BLyS receptors, TACI is required for responses to TI antigens. However, unlike classical TI antigens (e.g. polysaccharides), which never had the capacity to elicit classical T cell help via MHC restricted antigen presentation, peptides derived from PsaA in the PsaA-BLyS fusion should still retain this capacity, and should therefore be influenced by the immunological mechanisms involved in TD responses, such as the involvement of DC in the priming of the immune response. If PsaA-BLyS is indeed a TI antigen, we would still have to explain other features of the Ig response elicited by it, such as the boostability of the response, which is not observed with classical TI antigens like polysaccharides, but is a hallmark of TD responses. Additional indirect support for our assumption that T cells are critical to the immune response to PsaA, whether fused to BLyS or not, comes from the recent finding that polymerized bacterial flagellin, a protein which was long held to be a T-independent antigen has instead been found to be a T dependent antigen (C.J. Sanders, Y. Yu, D.A. Moore, 3rd, I.R. Williams, and A.T. Gewirtz, Humoral immune response to flagellin requires T cells and activation of innate immunity. J Immunol 177 (2006) 2810-18; PMID 16920916).

For these reasons, we feel that the discussion of TACI expression on DC is pertinent because it would be surprising (and very possibly a first, since flagellin should now be considered a TD antigen) if there were no contribution of T cells to the response to an exogenous protein antigen (PsaA-BLyS). Although CD4^+ ^T cell help for antibody responses to some viral antigens may be minimal, these antigens differ from PsaA-BLyS in that they are primarily endogenously synthesized and largely presented on MHC class I.

It would also be equally surprising if B cells were found to be the primary APC responsible for initiating the PsaA-BLyS response. This is underscored by the observation that one of the mechanisms for inducing tolerance to antigens is to exploit Ag presentation by B cells, both activated and non-activated.

In this regard, our discussion of TACI expression by DC represents the more conservative viewpoint (namely that DC are the primary APC for productive responses to TD antigens, and that virtually all proteins that have been studied to date thus far are invariably TD antigens).

We did not mention the role of TACI in class switching in the interest of focusing more directly on the observation that there was no PsaA-specific antibody elicited in response to PsaA-BLyS immunization in the absence of TACI. We certainly appreciate the reported role of TACI in immunoglobulin class switching but we did not perform any specific experiments to address this in our current studies. We do however now mention in the Discussion (p. 23) that BLyS mediates class switching, and the corresponding reference cited specifically states that TACI and BAFF-R are involved in antibody isotype switching.

In summary, we acknowledge that the answer to the TD/TI nature of the response to PsaA-BLyS is an important issue, but we will have to address it in future studies. Because we are prepared for the possibility that both TI and TD mechanisms are involved in the response to PsaA-BLyS, experiments using T cell deficient mice (to study the TI question) would have to be carried out in conjunction with experiments using animals with defects in pathways known to affect TD responses (CD40-CD40L axis, OX40-OX40L axis, etc.), as well as a robust means to compare the responses elicited across the various experimental cohorts in order to obtain satisfactory resolution to this question.

Yes indeed. We have stated in the Discussion that unambiguous instances of B cells being able to prime naïve T cells for a productive immune response are rare. The role of B cells in the BLyS-mediated response (other than producing antibodies) can only be addressed meaningfully after further experimentation to define the APC involved, the TD vs. TI nature of the response, etc.

We have deleted the last paragraph from the conclusion, as we have not supplied experimental data on BLyS-mediated enhancement of responses to TI antigens, and to avoid confusion.

We appreciate the positive comments on the manuscript and also appreciate the highly insightful observations and questions posed in this review.

## Competing interests

The authors declare that they have no competing interests.

## Authors' contributions

DOG designed and purified the recombinant proteins, performed *in vitro *assays, and drafted the manuscript, XD, performed protein purifications, immunizations and *in vitro *assays, QL performed immunizations and *in vitro *assays, DS performed in vitro assays, SSM performed *in vitro *assays, RJB developed research materials and wrote the manuscript, and NSG conceived of and designed the study, managed data collection, interpreted results and wrote the manuscript. All authors read and approved the final manuscript.
